# Transcriptome Analysis Reveals Key Pathways and Genes Involved in Lodging Resistance of Upland Cotton

**DOI:** 10.3390/plants13243493

**Published:** 2024-12-13

**Authors:** Yuan Wang, Ao Feng, Caiwang Zhao, Xiaomei Ma, Xinyu Zhang, Yanjun Li, Jie Sun

**Affiliations:** 1 The Key Laboratory of Oasis Eco-Agriculture, Agriculture College, Shihezi University, Shihezi 832003, China; wangyuan1@stu.shzu.edu.cn (Y.W.); fengao@stu.shzu.edu.cn (A.F.); 2023310@ouc.edu.cn (C.Z.); zhxy@shzu.edu.cn (X.Z.); 2 Cotton Research Institute, Xinjiang Academy of Agricultural and Reclamation Science, Shihezi 832000, China; maxm_09@163.com

**Keywords:** cotton, lodging, morphological and physiological characteristics, lignin, transcriptome, VIGS

## Abstract

Lodging resistance is one of the most important traits of machine-picked cotton. Lodging directly affects the cotton yield, quality and mechanical harvesting effect. However, there are only a few reports on the lodging resistance of cotton. In this study, the morphological and physiological characteristics and transcriptome of two upland cotton varieties with different lodging resistance were compared. The results showed that the stem strength; the contents of lignin, soluble sugar and cellulose; and the activities of several lignin biosynthesis-related enzymes of the lodging-resistant variety M153 were significantly higher than those of the lodging-susceptible variety M5330. Transcriptomic analysis showed that the expression level of several genes related to lignin, cellulose, starch and sucrose synthesis, and photosynthesis were significantly up-regulated in the lodging-resistant variety M153, which was consistent with the content determination results of lignin, cellulose and soluble sugar. Silencing two lignin biosynthesis-related genes (*GhPAL* and *Gh4CL*) in cotton via VIGS (Virus-Induced Gene Silencing) resulted in reduced lignin content and decreased lodging resistance in cotton. These results suggested that lignin, cellulose and soluble sugar contents were positively correlated with the lodging resistance of cotton, and lignin, cellulose and soluble sugar biosynthesis-related genes can be used as potential targets for improving the lodging resistance of cotton. These findings provide a theoretical basis for the cultivation of cotton varieties with strong lodging resistance in the future.

## 1. Introduction

Cotton is one of the world’s most important cash crops. China is one of the largest and leading cotton producers in the world [1]. As the largest cotton-producing area in the country, Xinjiang’s comprehensive mechanization rate of cotton has been increasing in recent years (https://www.thepaper.cn/newsDetail_forward_12231945 (accessed on 4 January 2024). The machine-picked cotton area continues to expand in Xinjiang, and mechanical harvesting has become the main method of cotton harvesting. Cultivating varieties suited to mechanical picking is essential for meeting the region’s mechanized production demands [2]. One critical agronomic challenge affecting cotton production is lodging, which can significantly reduce crop yield and quality, leading to notable economic losses [3]. Lodging resistance is a plant’s ability to withstand lodging, or the bending and toppling of stems [4]. This issue is significant in crops like rice, wheat, maize and barley, especially under challenging conditions such as strong winds, heavy rains, or high planting density [5,6,7,8]. Lodging can greatly reduce crop yield and quality by limiting light exposure, disrupting nutrient uptake and increasing disease susceptibility. In cotton, lodging resistance is a key trait that supports higher yields and facilitates efficient mechanical harvesting. Strong lodging resistance ensures the stems remain upright, allowing the cotton to enter the mechanical picking head smoothly, whereas lodging-prone stems hinder this process, reducing the harvest efficiency and overall yield.

Crop lodging can be divided into two main phenotypes. The first is stem lodging, which refers to the soft main stem and long middle and upper nodes of the plant. Due to the tall plant height and poor mechanical organization, crops are susceptible to lodging after being blown by strong winds [9]. The second is root lodging, which is caused by poor root growth and development, improper irrigation, and wind and rain during crop cultivation [10]. In cotton, lodging depends to a considerable extent on the stem anatomy [11]. Cotton lodging mostly involves stem lodging based on field practice. Although cotton lodging did not have a significant adverse effect on its yield in the past, the rapid development of mechanical harvesting requires cotton varieties to have strong lodging resistance.

The lodging resistance of crop stems is influenced by external factors such as cultivation, tillage, pests and diseases, as well as internal factors such as stem morphology and physiological characteristics [3,12]. The morphological characteristics of stems mainly include plant height, stem weight, stem diameter, and internode length and thickness [13,14,15]. For the physiological characteristics, the contents of lignin, cellulose and soluble sugars in stems have important roles in enhancing stem mechanical strength [16,17,18]. There is a significant or extremely significant positive correlation between the content of these substances and stem mechanical strength [19]. Stems’ mechanical characteristics represent an important aspect for evaluating crop lodging resistance [20]. The study of the mechanical characteristics of stems includes multiple aspects, such as puncture resistance, breaking resistance, and bending resistance, which represent the toughness of stems [18,21]. Stem mechanical strength has been found to be significantly positively correlated with stem lodging resistance [22].

At present, research on lodging resistance is mostly focused on crops such as wheat, corn and rice [6,7,23]. However, there are still few reports on the lodging resistance of cotton. In this study, two cotton varieties with different lodging resistance were used as experimental materials to analyze the differences in their morphological characteristics and physiological characteristics. Additionally, transcriptomic sequencing was used to identify the differential pathways and differentially expressed genes (DEGs) between lodging-resistant and lodging-susceptible varieties. Our results will lay the foundation for the cultivation and selection of lodging-resistant cotton varieties and provide candidate genes for using genetic engineering to breed new varieties of lodging-resistant cotton.

## 2. Results

### 2.1. Stem Morphological Characteristics of Cotton Varieties with Different Lodging Resistance

**Figure 1:**
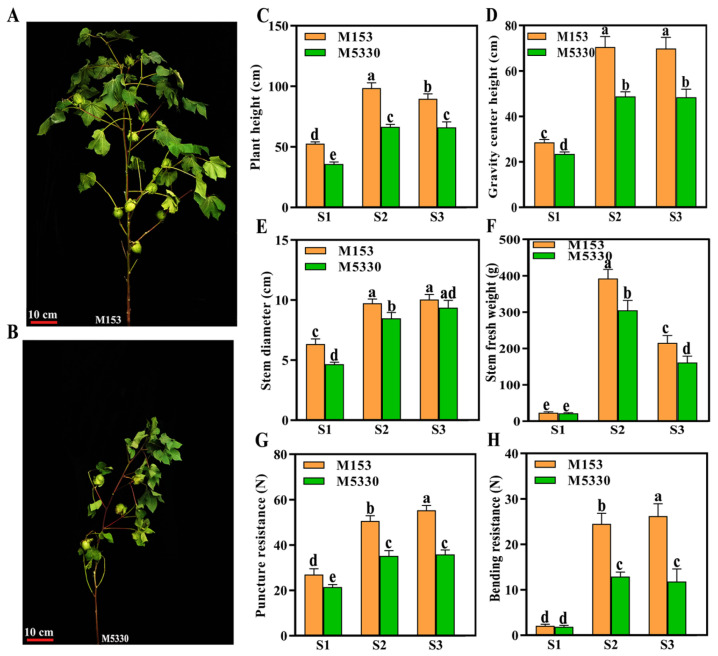
Images and the morphological indices of two cotton varieties at three developmental stages. (**A**) An observation of stem lodging of M153 cotton at the boll stage. (**B**) An observation of stem lodging of M5330 cotton at the boll stage. (**C**) Plant height. (**D**) Gravity center height. (**E**) Stem diameter. (**F**) Stem fresh weight. (**G**) Puncture resistance of the stem. (**H**) Bending resistance of the stem. S1: the bud stage; S2: the boll stage; S3: the boll opening stage. SPSS 26.0 software was used for statistical analysis of the data. Each value is the mean of twenty biological replicates. Duncan‘s new multiple range method was used to test the significance of the differences. Different lowercase letters represent statistically significant differences among varieties in the same growing stage at the *p* < 0.05 level from one-way ANOVA tests.

Two upland varieties with different lodging resistance were used in this study. It was observed that the stem of M153 was straight throughout the developmental process, while the stem of many M5330 plants began to bend from the boll stage (Figure 1A,B). The measure of morphological indices showed that the plant height, gravity center height, stem diameter and stem fresh weight of M153 were higher than that of M5330 (Figure 1C–F). The stem mechanical strength of the two varieties were then measured. The results showed that the value of stem puncture and bending resistance strength of M153 gradually increased from the bud stage to the boll opening stage, while those of M5330 increased from the bud stage to the boll stage, with no significant difference between the boll stage and the boll opening stage (Figure 1G,H). Additionally, the puncture resistance and bending resistance strength of M153 were significantly higher than those of M5330 at all three stages (Figure 1G,H). It is generally considered that taller plants are susceptible to lodging, while shorter ones are less susceptible to lodging [5]. Our results showed that M153 has strong lodging resistance, despite having a taller plant height. M5330 is susceptible to lodging, despite being shorter.

### 2.2. Lignin Deposition, Lignin Content and Lignin Biosynthesis-Related Enzyme Activity

**Figure 2:**
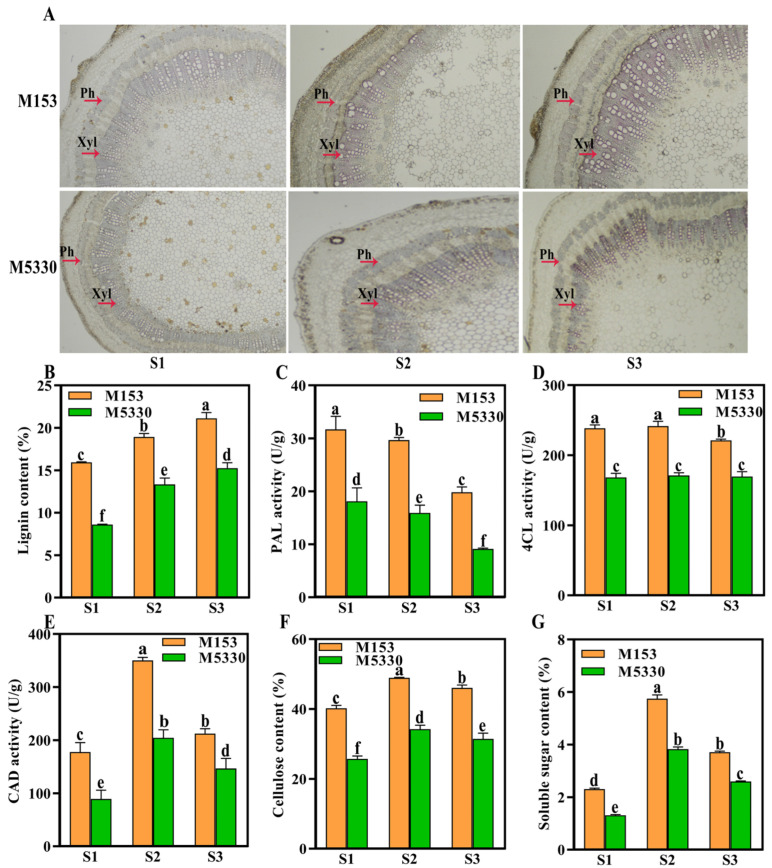
Histochemical staining and physiological indices of the two cotton varieties at three developmental stages. (**A**) Lignin deposition of the stems of two cotton varieties at three developmental stages following phloroglucinol staining. (**B**) Lignin content. (**C**) PAL activity. (**D**) 4CL activity. (**E**) CAD activity. (**F**) Cellulose content. (**G**) Soluble sugar content. The positions indicated by the red arrows are the phloem (Ph) and xylem (Xyl). SPSS 26.0 software was used for statistical analysis of the data. Each value is the mean of ten biological replicates. Duncan‘s new multiple range method was used to test the significance of the differences. Different lowercase letters represent statistically significant differences among varieties in the same growing stage at the *p* < 0.05 level from one-way ANOVA tests.

Lignin can provide structural support for the plant cell wall, increase cell strength, and play an important role in strengthening stem strength [24]. Therefore, we first compared the lignin deposition and lignin content in the stems of the two varieties. The lignin deposition was observed using histochemical staining. At all three stages, lignin was mainly deposited in the phloem (Ph) and xylem (Xyl) of the stems of the two varieties. The fuchsia color in the stem gradually deepened with stem development. The stained color did not show significant differences between the two varieties at all three stages. It was notable that M153 presented a greater stained area in the xylem compared to M5530 at the boll opening stage (S3) (Figure 2A). The total lignin content of the two varieties increased with stem development, and M153 had significantly higher lignin content than M5530 at all three stages (Figure 2B), indicating that the degree of lignin deposition and the lignin content in M153 were greater than those in M5530.

The activity of three lignin biosynthesis-related enzymes, including PAL, 4CL and CAD, was then analyzed. The PAL activity in the two varieties decreased and the CAD activity first increased and then decreased, while the 4CL activity did not change with stem development (Figure 2C–E). The four enzymes’ activities in M153 were significantly higher than those in M5530 at all three stages (Figure 2C–E).

### 2.3. Cellulose and Soluble Sugar Content in Cotton

Cellulose is an important component of the plant cell wall and plays an important role in maintaining the morphology of plant cells [25]. Soluble sugars are non-structural carbohydrates stored in stems, and play an important role in maintaining stem strength [26]. The cellulose and soluble sugar contents were therefore determined. The cellulose and soluble sugar contents in both varieties first increased and then decreased with stem development, and the lodging-resistant variety M153 had significantly higher cellulose and soluble sugar contents than the lodging-susceptible variety M5530 at all three stages (Figure 2F,G).

### 2.4. RNA-Aseq and Differential Expression Analysis

**Figure 3:**
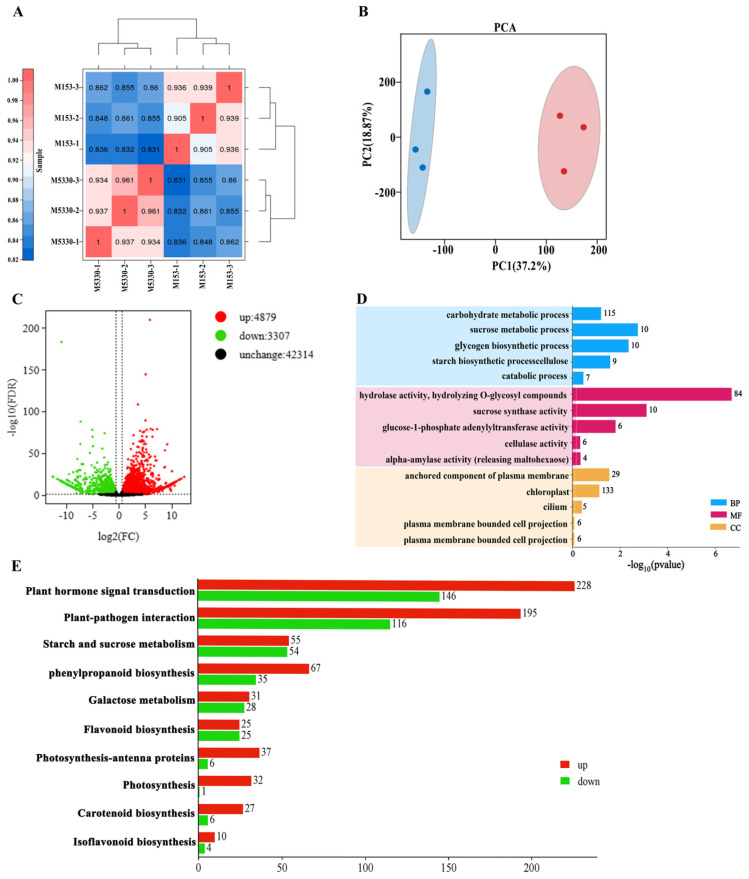
Transcriptome analysis of two cotton varieties at the boll stage. (**A**) A heatmap of expression level correlation for pairwise samples. (**B**) Principal component analysis (PCA) of gene expression between sample groups. (**C**) A volcano plot of DEGs. Red and green points represent the up-regulated and down-regulated DEGs in the lodging resistance variety, respectively. (**D**) Go analysis of DEGs. (**E**) KEGG analysis of DEGs.

To explore some genes responsible for the lodging resistance of cotton, we conducted RNA-seq analysis on the stems of two varieties. The stems of many M5330 plants began to bend at the boll stage, while the stems of all M153 plants were straight, indicating significant differences in lodging phenotypes between the two varieties from the boll stage onwards. Therefore, the stems at the boll stage of the two varieties were selected for RNA-seq analysis, and a total of six RNA-seq libraries (M153-1, M153-2, M153-3, M5330-1, M5330-2 and M5330-3) were constructed. A total of 36.21 Gb clean data were generated, and the percentage of bases in Q30 was 93.73% and above. The comparison efficiency of reads between each sample and the reference genome ranged from 92.87% to 95.87% (Table 1). The Pearson correlation analysis and principal component analysis indicated a good level of repeatability of the RNA-seq results (Figure 3A,B).

The differentially expressed genes (DEGs) between the lodging-resistant variety M153 and the lodging-susceptible variety M5330 were identified by using the criteria of |fold change| ≥ 1.5 and False Discovery Rate (FDR) < 0.05. It was found that there were 8186 DEGs between M153 and M5330, of which 4879 were up-regulated and 3307 were down-regulated in the lodging-resistant variety M153 (Figure 3C). The expression levels of nine randomly selected genes were analyzed by using qRT-PCR, and the results were largely consistent with RNA-seq data, indicating that the RNA-seq results were reliable (Figure S1).

### 2.5. GO and KEGG Analysis

To further explore the function of these DEGs, GO enrichment analysis and KEGG pathway analysis were conducted on all identified DEGs. In the biological process category, the sucrose metabolic process, glycogen biosynthetic process and starch biosynthetic process were the top three significantly enriched terms. In the molecular function category, the hydrolase activity of hydrolyzing O-glycosyl compound was the most significantly enriched term, followed by sucrose synthase activity. In the component category, the anchored component of the plasma membrane and chloroplasts were the top two significantly enriched terms (Figure 3D). KEGG pathway analysis showed that DEGs were enriched in plant hormone signal transduction, plant–pathogen interaction, starch and sucrose metabolism, phenylpropanoid biosynthesis, galactose metabolism, flavonoid biosynthesis, photosynthesis-antenna proteins and photosynthesis pathways (Figure 3E).

### 2.6. DEGs Related to Phenylpropanoid Biosynthesis Pathway

**Figure 4:**
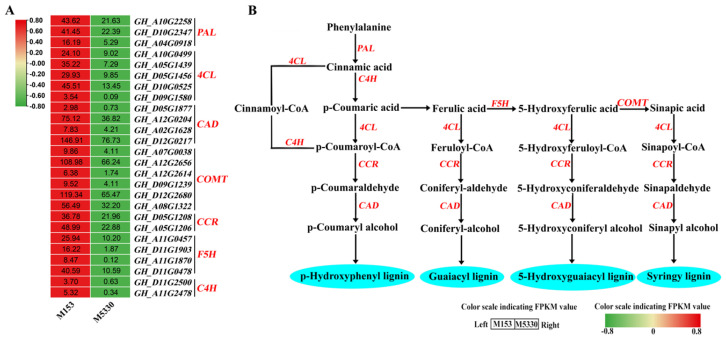
Transcript profiling of genes in phenylpropanoid biosynthetic pathway in two cotton varieties. (**A**) Heatmap of DEGs involved in lignin biosynthesis pathway in two cotton varieties. (**B**) Diagram of phenylpropanoid biosynthesis pathway.

The phenylpropanoid biosynthesis pathway serves as a rich source of metabolites in plants, being required for the biosynthesis of lignin [27]. A total of 102 DEGs were found in the phenylpropanoid pathway, including 67 up-regulated DEGs and 35 down-regulated ones. The 67 up-regulated DEGs harbored several lignin synthesis-related genes (Table S2), mainly including three *PAL* (phenylalanine ammonia lyase), five *4CL* (4-coumarate: CoA ligase), four *CAD* (cinnamy-alcohol dehydrogenase), six *COMT* (catechol-o-methyltransferase), two *CCR* (cinnamoyl-CoA reductase), four *F5H* (Ferulate 5-hydroxylase) and two *C4H* (Cinnamate 4-hydroxylase) genes. The heat map shows that the expression level of these lignin synthesis-related genes in M153 were significantly higher than that in M5330, which was consistent with the results of lignin content determination (Figure 4A). From the diagram (Figure 4B), it can be seen that lignin synthesis-related genes are up-regulated along the route flowing towards three lignin monomers. The up-regulation of these lignin biosynthesis-related genes in M153 likely contributes to the higher lignin content observed in the lodging-resistant variety, thereby enhancing its lodging resistance.

### 2.7. DEGs Related to Photosynthesis and Starch and Sucrose Metabolism Pathways

**Figure 5:**
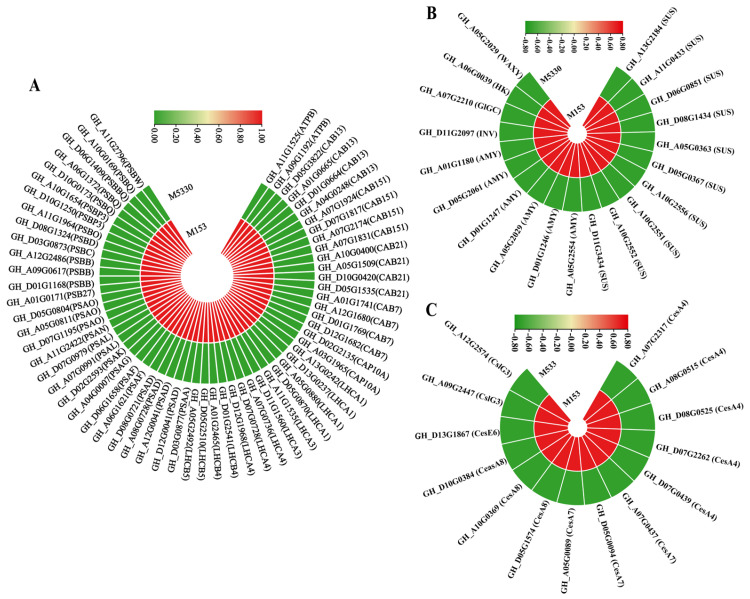
A heatmap of DEGs related to the photosynthesis and starch and sucrose metabolism pathways and cellulose biosynthesis. (**A**) A heatmap of DEGs related to the photosynthesis pathway. (**B**) A heatmap of DEGs related to the starch and sucrose pathways. (**C**) A heatmap of DEGs related to cellulose biosynthesis.

It is well-known that photosynthesis is the process in which plants produce sugars. Several photosynthesis-related genes were up-regulated in the lodging-resistant variety M153 (Table S3), mainly including 30 photosystem II genes (*LHCA1*, *LHCA3*, *LHCA4*, *PSAA*, *PSAD*, *PSAF*, *PSAG*, *PSAK*, *PSAL*, *PSAN*, *PSAO*, *PSBB*, *PSBC*, *PSBD* and *PSBO*), 12 photosystem I genes (*PSBP3*, *PSBQ*, *PSBW*, *PSB27*, *LHCB4* and *LHCB5)*, and 18 chlorophyll a-b binding protein genes (*CAB13*, *CAB151*, *CAB21*, *CAB7* and *CAP10A*) (Figure 5A). Additionally, the expression levels of 55 genes in the starch and sucrose metabolism pathway closely related to photosynthesis were significantly up-regulated in the lodging-resistant variety M153 (Table S4), mainly including 10 sucrose synthase (*SUS*), 6 alpha-amylase genes (*AMY*) and 1 invertase (*INV*) genes (Figure 5B). Sucrose synthase (*SUS*) is a key enzyme in plant sugar metabolism, as it can catalyze the reversible cleavage of the photosynthetic end-product sucrose into fructose and UDP-glucose. Alpha-amylase can catalyze the hydrolysis of starch into smaller carbohydrate molecules, such as glucose [28]. The expression of genes related to the photosynthesis and starch and sucrose pathways in M153 were significantly higher than that in M5330, which was consistent with the results of soluble sugar content determination. The up-regulation of genes related to photosynthesis and starch and sucrose metabolism in M153 likely contributed to the higher soluble sugar content observed in the lodging-resistant variety.

### 2.8. DEGs Related to Cellulose Synthesis

Cellulose is the main polymer that constitutes the primary and secondary cell walls [29], and it is considered to provide a framework for the assembly of other cell wall polymers, including hemicellulose, pectin and lignin, which maintain the morphology of plant cells and support the uprightness of plants [25]. In this study, 14 cellulose synthase genes were found to be up-regulated in the lodging-resistant variety M153 (Table S5), which was consistent with the results of cellulose content determination (Figure 5C). Taken together, the up-regulation of cellulose synthesis genes in M153 likely contributes to the higher cellulose content observed in the lodging-resistant variety, thereby reinforcing its resistance to lodging.

### 2.9. Functional Confirmation of Two DEGs Related to Lignin Synthesis by VIGS

**Figure 6:**
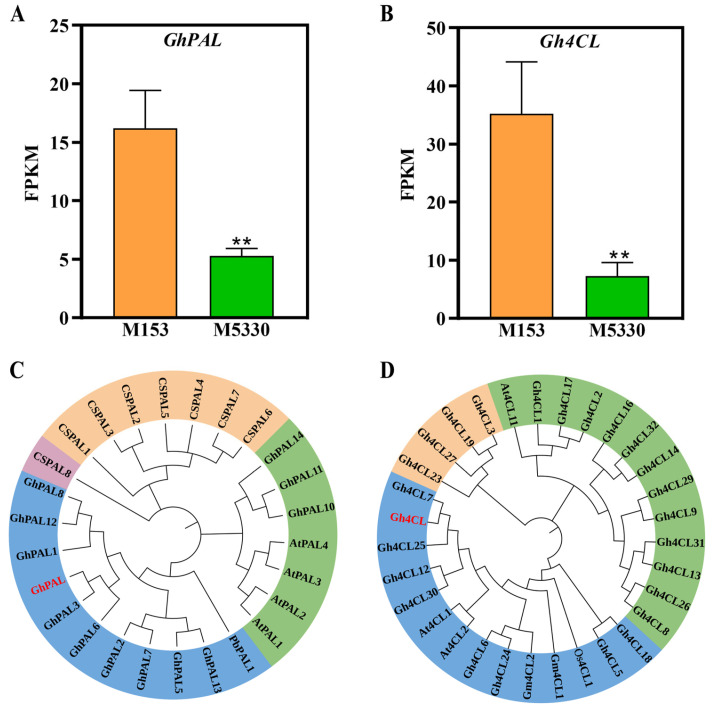
The expression levels and phylogenetic tree analysis of *GhPAL* and *Gh4CL*. (**A**) The expression level of the *GhPAL* gene in the two cotton varieties. (**B**) The expression level of the *Gh4CL* gene in the two cotton varieties. (**C**) A phylogenetic tree of GhPAL and PAL from other plants. (**D**) A phylogenetic tree of Gh4CL and PAL from other plants. GhPAL and Gh4CL marked with red are genes used for functional confirmation by HIGS. Gh: *Gossypium hirsutum*; Pb: *Pyrus bretschneideri*; At: *Arabidopsis thaliana*; Cs: *Cucumis sativus*; Gm: *Glycine max*; Os: *Oryza sativa.* The phylogenetic tree was constructed using MEGA11.0 via the N-J method. Each value is the mean of three biological replicates. Statistical analysis of the data was conducted using IBM SPSS Statistics version 26.0, with statistical significance being assessed via Student’s *t*-test. ** represents a significant difference at *p* < 0.01 between the two cotton varieties.

PAL and 4CL enzymes play important roles in lignin biosynthesis [30]. Through transcriptome data analysis, we found that several lignin biosynthesis-related genes showed higher expression levels in the lodging-resistant variety (Figure 4). Of these genes, the expression levels of the *GhPAL* (GH_A04G0918) and a *Gh4CL* genes (GH_A05G1439) showed more significant changes compared to the other genes, exhibiting 5-fold and 6-fold up-regulation in the lodging-resistant variety, respectively (Figure 6A,B). The phylogenetic tree analyses showed that *GhPAL* was clustered into one branch with *PbPAL1*, which has been reported to be involved in lignin biosynthesis [31], and *Gh4CL* was clustered into the Class I subfamily with *At4CL1*, *At4CL2* and *Gm4CL1*, which have been reported to be associated with lignin biosynthesis (Figure 6C,D) [32,33], suggesting that *GhPAL* and *Gh4CL* may be involved in lignin biosynthesis in cotton. Therefore, we selected the two genes for VIGS functional verification.

**Figure 7:**
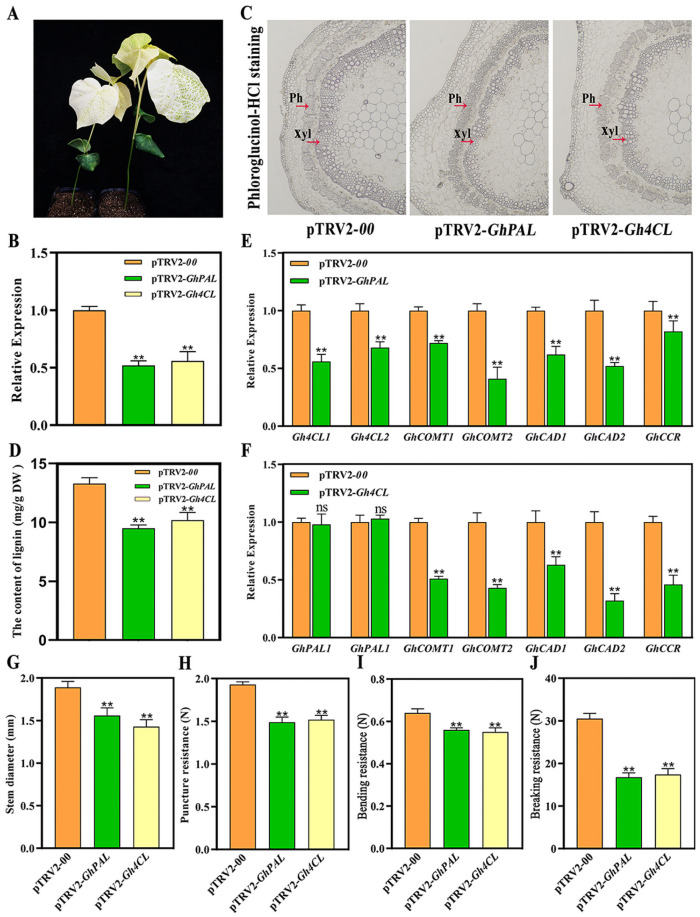
The morphological and physiological indices of cotton after silencing the of *GhPAL* and *Gh4CL* by VIGS. (**A**) The bleaching phenotype of pTRV2-*GhCHLI*-treated cotton plants. (**B**) The relative expression level of *GhPAL* in pTRV2:*GhPAL*-treated plants and *Gh4CL* genes in pTRV2:*Gh4CL*-treated plants. (**C**) The lignin deposition in stems of pTRV2-*GhPAL*- and pTRV2-*Gh4CL*-treated plants following histochemical staining. Ph: phloem; Xyl: xylem. (**D**) The lignin content in the pTRV2-*GhPAL*- and pTRV2-*Gh4CL*-treated cotton plants. (**E**) The expression levels of genes related to lignin biosynthesis in pTRV2-*00*- and pTRV2-*GhPAL*-treated cotton plants. (**F**) The expression levels of genes related to lignin biosynthesis in pTRV2-*00*- and pTRV2-*Gh4CL*-treated plants. (**G**) The stem diameter of VIGS-treated plants. (**H**) The puncture resistance strength of VIGS-treated plants. (**I**) The bending resistance strength of VIGS-treated plants. (**J**) The breaking resistance strength of VIGS-treated plants. Each value is the mean of three biological replicates in figure (**B**,**D**,**E**,**F**), and the mean of ten biological replicates in figure (**G**–**J**). Statistical analysis of the data was conducted using IBM SPSS Statistics version 26.0, with statistical significance being assessed via Student’s *t*-test. ** represents a significant difference at *p* < 0.01 between VIGS-treated plants (pTRV2-*GhPAL*- and pTRV2-*Gh4CL*-treated plants) and pTRV2-*00*-treated plants and the ns represents no significant difference.

The pTRV2-*00*-treated plants were used as a negative control, and the pTRV2-*GhCHLI*-treated plants were used as a positive control. After 10 days of injection, when the leaves of pTRV2-*GhCHLI*-treated plants showed the bleaching phenotype (Figure 7A), the total RNA of the stem tissues from pTRV2-*GhPAL*- and pTRV2-*Gh4CL*-treated plants were extracted and reverse-transcribed into cDNA for gene silencing efficiency detection. The results showed that the expression levels of *GhPAL* in pTRV2-*GhPAL*-treated plants, and *Gh4CL* in pTRV2-*Gh4CL*-treated plants, were significantly lower than those in pTRV2-*00*, indicating that the expression of *GhPAL* and *Gh4CL* genes had been successfully inhibited (Figure 7B).

To explore whether silencing *GhPAL* and *Gh4CL* genes has an effect on lignin biosynthesis, we compared the lignin deposition and lignin content in the stems of the pTRV2-*00*- and VIGS-treated plants. Histochemical staining showed that pTRV2-*GhPAL*- and pTRV2-*Gh4CL*-treated plants presented a smaller stained area and a lighter color compared to pTRV2-*00*-treated plants (Figure 7C). The total lignin content of pTRV2-*GhPAL*- and pTRV2-*Gh4CL*-treated plants was significantly lower than that of pTRV2-*00*-treated plants, indicating that the degree of lignin deposition and the lignin content in VIGS-treated plants were lower than those in pTRV2-*00*-treated plants (Figure 7D). In addition, we examined the expression levels of several lignin biosynthesis-related genes and found that the expression levels of *Gh4CL1*, *Gh4CL2*, *GhCOMT1*, *GhCOMT2*, *GhCAD1*, *GhCAD2* and *GhCCR* were significantly reduced in pTRV2-*GhPAL*-treated plants compared to the pTRV2-*00* control plants. In pTRV2-*Gh4CL*-treated plants, the expression levels of all of the lignin biosynthesis-related genes were decreased except for *GhPAL1* and *GhPAL2* (Figure 7E,F).

To explore whether silencing *GhPAL* and *Gh4CL* genes has an effect on cotton lodging resistance, the morphological indices of VIGS-treated plants were investigated. The results showed that the stem diameter, puncture resistance, bending resistance and breaking resistance strength of pTRV2-*GhPAL*- and pTRV2-*Gh4CL*-treated plants were significantly lower than those of pTRV2-*00*-treated plants (Figure 7G–J), suggesting that silencing the *GhPAL* and *Gh4CL* genes can lead to reduced lodging resistance of cotton, and the two genes play important roles in the lodging resistance of cotton.

## 3. Discussion

A large number of studies have shown that plant height and gravity center height are negatively correlated with crop lodging resistance. For example, the lodging resistance of flax was negatively correlated with plant height and gravity center height [34]. Plant dwarfing could reduce the gravity center height, thus improving the lodging resistance of plants. In a sense, reducing plant height is the most effective measure to improve plant lodging resistance [35]. However, other studies found that dwarf varieties were not necessarily resistant to lodging, and high-stalk varieties were not necessarily susceptible to lodging [5]. In this study, the plant height and gravity center height of the lodging-resistant variety M153 were significantly higher than those of the lodging-susceptible variety M5330. However, M153 was resistant to lodging, while M5330 was prone to lodging, indicating that the stem of M153 was stronger than that of M5330, which was confirmed by the results of puncture and bending resistance strength determination. To find the genes responsible for the lodging resistance of cotton, these two varieties were selected for further research.

Stem mechanical strength is a complex trait that is affected by many factors, including the internal structure and chemical composition of the stem [12,36]. Many studies have found that there is a strong correlation between stem mechanical strength and lignin content. In sudangrass [37], chrysanthemum [38], kodo millet [39] and rice [40], a decrease in lignin content has been found to lead to a decrease in stem mechanical strength. Lignin biosynthesis is closely related to the activities of several related enzymes. In common buckwheat, lignin content was significantly positively correlated with PAL, 4CL and CAD activities [41]. In herbaceous peony, the total lignin content and the activities of 4CL, PAL and CAD were higher in the upright variety than in the bending variety during stem development [3]. In this study, the lignin content and the PAL, 4CL and CAD activities in the lodging-resistant variety M153 were significantly higher than those of the lodging-susceptible variety M5330 during stem development, which was consistent with previous reports.

Lignin content is also closely related to the expression level of lignin biosynthesis-related genes. In herbaceous peony, ten lignin biosynthesis-related genes, including 26 members, were found to be closely associated with lignin content [3]. The up-regulated expression of *4CL7* in cotton led to lignin accumulation [42]. In melon, the up-regulation of *PAL*, *C4H* and *4CL* promoted lignin biosynthesis [43]. In this study, silencing two lignin biosynthesis-related genes (*GhPAL* and *Gh4CL*) resulted in reduced lignin content of cotton. The cotton genome contains several *PAL* and *4CL* genes organized into families, but the functions of individual gene members within these families can differ. For instance, within the *4CL* gene family, some genes may be involved in lignin biosynthesis [42,43], while others participate in the biosynthesis of flavonoids or other compounds [42]. In this study, transcriptome comparisons between lodging-resistant and lodging-susceptible varieties identified potential lignin biosynthesis-related genes that were significantly up-regulated in the lodging-resistant variety. Based on this analysis, we selected the *GhPAL* (GH_A04G0918) and *Gh4CL* (GH_A05G1439) genes that were significantly up-regulated in the lodging-resistant variety and conducted functional validation through VIGS to investigate their roles in lignin biosynthesis and their impact on cotton lodging resistance. The results demonstrated that *GhPAL* (GH_A04G0918) and *Gh4CL* (GH_A05G1439) are indeed involved in lignin biosynthesis, supporting their contribution to the improved lodging resistance observed in the M153 variety. This key finding underscores the importance of targeted gene selection in studies of lignin biosynthesis and cotton lodging resistance. Due to the upstream position of the *GhPAL* and *Gh4CL* genes in the lignin biosynthesis pathway, their silencing leads to a decrease in lignin content and the down-regulation of downstream genes (*GhCOMT*, *GhCAD* and *GhCCR*) in VIGS-treated plants.

The accumulation of carbohydrates such as sucrose and polysaccharide in the stems of lodging-resistant varieties promoted the synthesis of lignin, cellulose and hemicellulose [19]. The network structure formed by the interweaving of macromolecular compounds could enhance the elasticity of the stem wall and improve the lodging resistance of plants [44]. Soybean plants under shade stress will cause a decrease in soluble sugar content and reduce the synthesis of lignin substrates, thus weakening the stem strength of soybean [45]. The accumulation of carbohydrates such as sucrose and polysaccharides in rice stems promoted the synthesis of lignin, enhanced stem strength, and improved plant lodging resistance [16]. In this study, we found that the soluble sugar content in the lodging-resistant variety M153 was higher than that in the lodging-susceptible variety M5330. Furthermore, RNA-seq data showed that several genes related to photosynthesis and the starch and sucrose metabolic pathways were up-regulated in M153 compared to M5330. This aligns with the higher soluble sugar content observed in M153, suggesting that these genes contributed to the increased sugar levels and enhanced lodging resistance in the lodging-resistant variety.

Cellulose is an important component of the plant cell wall and plays an important role in maintaining cell morphology and plant uprightness [25]. In rice and wheat, an increase in cellulose content has been found to enhance crop lodging resistance [17]. The synthesis of cellulose is regulated by a variety of genes, among which the cellulose synthase (*CESA*) and cellulose synthase (*CSL*) genes play important roles in the process of cellulose synthesis. Studies have shown that the mutation of *CESA9* had an effect on the integrity of the *CESA4/7/9* complex, resulting in the degradation of CESA protein in cellulose biosynthesis, thereby reducing the cellulose content and lodging resistance of rice [46]. Three cellulose synthase genes, *AtCesA4*, *AtCesA7* and *AtCesA8*, related to secondary wall synthesis were identified from *Arabidopsis thaliana*. After knocking out these three genes, the synthesis of cellulose was reduced and the stem strength was weakened [47]. In this study, RNA-seq data revealed that several *CesA* genes were significantly up-regulated in the lodging-resistant variety M153 compared to the lodging-susceptible variety M5330. This up-regulation corresponds to the higher cellulose content observed in M153, suggesting that the increased expression of these CesA genes may have contributed to the elevated cellulose levels, thereby enhancing lodging resistance in the M153 variety.

## 4. Materials and Methods

### 4.1. Plant Materials and Culture Conditions

Two upland cotton varieties with different lodging resistance, M153 (lodging-resistant variety) and M5330 (lodging-susceptible variety), were used in this study. The two varieties were provided by the Cotton Research Institute of Shihezi University. For field planting, the two varieties were planted in the experimental field of the cotton research institute of Shihezi University (44°32′ N, 86°08′ E) in Xinjiang. The morphological indices were determined at the bud stage, boll stage and boll opening stage. The second internodes of the stems at the three stages were collected and used for mechanical strength and physiological index determination. For indoor planting, the seeds of M153 were grown in a plant incubator at 28 °C with a photoperiod of 16 h light/8 h dark and a relative humidity of 60%.

### 4.2. Morphological Indices and Mechanical Strength Determination

The plant height, the length of the second internode and the gravity height center (the distance from the base of the stalk to the balance fulcrum of the stem) were measured with a straight ruler. The stem diameter and stem weight were measured with a vernier caliper and a balance, respectively. The stem mechanical strength (puncture resistance and bending resistance) was measured with a stalk strength tester (Wenzhou Tripod Instrument Manufacturing, Wenzhou, China). The second internode was used for puncture resistance determination. The probe of the stalk strength tester was suspended and pulled down to a 45 degree angle with the ground to measure the bending resistance strength of the cotton plant according to the instructions.

### 4.3. Lignin, Cellulose and Soluble Sugar Content Measurements

The second internodes at the three stages were dried and ground into powder, and then sieved through a 40 mesh sieve. The contents of lignin were determined using a lignin content kit, the contents of cellulose were determined using a cellulose content kit, and the contents of sugar were determined using a soluble sugar content kit. All kits were from the Suzhou grace biotechnology company (Suzhou, China).

### 4.4. Lignin Deposition Observations

Lignin deposition observations were conducted using the method reported in [3]. Briefly, the second internodes at the three stages were fixed in 2.5% glutaraldehyde solution and FAA fix solution for lignin deposition observations. The prepared paraffin sections of stem were dewaxed to water and stained with phloroglucinol solution for 2 min, and then sealed with 18% hydrochloric acid solution. The samples were observed and photographed under an optical microscope (CX31, Olympus, Tokyo, Japan) within 3 min.

### 4.5. Lignin Biosynthesis-Related Enzyme Activity Measurements

The fresh samples of stem at different stages were ground in a mortar to be homogenized to determine the activity of enzymes related to lignin synthesis (4CL, PAL, CAD). The 4CL activity was determined using a 4-coumarate coA ligase (4CL) activity assay kit, the PAL activity was determined using a phenylalnine ammonialyase (PAL) activity assay kit, and the CAD activity was determined using a cinnamyl alcohol dehydrogenase (CAD) activity assay kit. All kits were purchased from the Solarbio company (Beijing, China).

### 4.6. RNA Sequencing (RNA-Seq)

The second internode at the boll stage was quickly frozen in liquid nitrogen for RNA-seq analysis. The RNA was extracted using an RN40-EASYspin plant microRNA rapid extraction kit (Aidlab, Beijing, China). The concentration and purity of the extracted nucleic acid were detected using a Nanodrop2000 spectrophotometer (Thermo Scientific, Waltham, MA, USA), and the integrity of RNA was detected using an Agient2100 biological analyzer (Agilent Technologies, Santa Clara, CA, USA). Using 1 ug RNA sample, according to the manufacturer’s instructions, a sequencing library was generated using a Hieff NGS Ultima Dual-mode mRNA Library Prey Kit for lllumina (Yeasen Biotechnology (Shanghai) Co., Ltd., Shanghai, China). The quality of the sequencing libraries was evaluated on an Agilent Bioanalyzer 2100 system. Subsequently, sequencing was performed on an lllumina NovaSeq platform, and the raw data were processed by BMKCloud (www.biocloud.net (accessed on 24 April 2024). Sequences of low quality and connectors were removed from the raw data to obtain clean data by using Perl scripts. The clean data were aligned with the cotton reference genome (TM-1) (http://cotton.zju.edu.cn/ (accessed on 27 April 2024) to obtain mapped data by using HISAT2 (v2.0.4). Differentially expressed genes were screened using DESeq2_EBSeq (v1.6.3) with the criteria of |fold change| ≥ 1.5 and False Discovery Rate (FDR) < 0.05. KEGG (Kyoto Encyclopedia of Genes and Genomes) enrichment analysis of differentially expressed genes (DEGs) was performed using R/clusterProfiler (v3.10.1), and the significance level was *p* value < 0.05 [48,49].

### 4.7. TRV (Tobacco Rattle Virus) Treatment

Total RNA was extracted from stems of the cotton variety M153 using an RN40-EASYspin plant microRNA rapid extraction kit (Aidlab, Beijing, China) and reverse-transcribed into cDNA using EasyScript^®^ One-Step gDNA Removal and cDNA Synthesis SuperMix (TransGen Biotech, Beijing, China). The full-length coding sequences of the *GhPAL* (GH_A04G0918) and *Gh4CL* (GH_A05G1439) genes were amplified with specific primers, GhPAL-F/R and Gh4CL-F/R, respectively. The purified PCR products were integrated into the pTRV2 vector with In Fusion enzymes (Vazyme, Nanjing, China) to generate the VIGS vectors pTRV2-*GhPAL* and pTRV2-*Gh4CL*, respectively, which were then transformed into the Agrobacterium tumefaciens strain GV3101 through electroporation. Agrobacterium suspensions harboring VIGS vectors were prepared and injected into cotton leaf according to previous reports [50]. When the pTRV2:*GhCHLI*-treated plants showed an obvious bleaching phenotype on their leaves, the stems of 10 pTRV2-*GhPAL*- and pTRV2-*Gh4CL*-treated plants were separately collected for lignin content determination and silence efficiency detection. The lignin content and deposition, stem puncture resistance, breaking resistance and bending resistance of VIGS-treated plants were determined using the methods mentioned above. Silence efficiency detection was conduced via qRT-PCR (quantitative real-time polymerase chain reaction), which was performed with GhPAL-F/R and Gh4CL-F/R as premiers, cDNA of M153 as a template and cotton *GhNBQ7* as the internal reference. The qRT-PCR was conducted using the Power SYBR Green PCR Master Mixture (Roche, Basel, Switzerland) on a Roche Light Cycler 480 II system (Roche, Basel, Switzerland). Relative gene expression levels were calculated using the 2DDCt method. All primers used in this study are shown in Table S1.

### 4.8. Gene Expression Analysis

RNA was extracted from cotton stems and reverse-transcribed into cDNA as mentioned above. The expression levels of several differentially expressed genes between the two varieties were determined via qRT-PCR. The specific primers used in qRT-PCR are listed in Table S1. Cotton *GhNBQ7* was used as the internal reference gene. The qRT-PCR assay and gene expression level calculation were conducted as mentioned above.

### 4.9. Statistical Data Analysis

SPSS 26.0 software was used for statistical analysis of the data. Regarding the results of the one-way analysis of variance (ANOVA) with Tukey’s multiple comparisons test, different letters indicate significant differences, with *p* < 0.05. Statistical analyses of the experimental groups including the controls were evaluated using a two-tailed Student’s *t*-test (**p < 0.01).

## 5. Conclusions

In summation, we combined morphological and physiological characteristics and transcriptomic analyses to compare the differences between lodging-resistant and lodging-susceptible cotton varieties. It was found that the contents of lignin, cellulose and soluble sugar were higher in the lodging-resistant variety M153 compared to the lodging-susceptible variety. Moreover, the expression levels of genes related to lignin, cellulose and soluble sugar biosynthesis were significantly higher in M153 than M5330, which was consistent with the results of lignin, cellulose and soluble sugar content determination. These results suggest that lignin, cellulose and soluble sugar contents were positively correlated with the lodging resistance of cotton, and lignin, cellulose and soluble sugar biosynthesis-related genes can be used as potential targets for improving the lodging resistance of cotton. These findings provide a theoretical basis for the cultivation of cotton varieties with strong lodging resistance in the future.

## Figures and Tables

**Table 1:** Quality control data statistics.

Samples	Clean Reads	% ≥ Q30	Comparison Efficiency	GC Content
M153-1	21,085,390	94.00%	94.56%	44.40%
M153-2	20,148,910	93.80%	92.87%	44.69%
M153-3	19,108,469	93.94%	95.68%	44.04%
M5330-1	20,972,364	93.88%	93.24%	44.83%
M5330-2	19,757,058	93.88%	95.82%	43.90%
M5330-3	19,994,421	93.73%	95.87%	44.04%

## Data Availability

Raw sequencing data can be accessed though https://www.ncbi.nlm.nih.gov/geo/ (accessed on 12 March 2024), the accession number PRJNA1170202.

## Supplementary Materials

The following supporting information can be downloaded at: https://www.mdpi.com/article/10.3390/plants13243493/s1, Figure S1. Analysis of RNA-seq data reliability. Table S1. Primer sequences used in this study. Table S2. List of the phenylpropanoid pathway genes. Table S3. List of the photosynthesis pathway genes. Table S4. List of the starch and sucrose pathways genes. Table S5. List of the cellulose synthesis genes.
