# Supplementary material for: Transcriptome Analysis Reveals Key Pathways and Genes Involved in Lodging Resistance of Upland Cotton
Source: Plants (Basel). 2024 Dec 13;13(24):3493. doi: 10.3390/plants13243493 (PMC11728647; doi:10.3390/plants13243493)
Supplement: Supplementary file 1 [file plants-13-03493-s001.zip › plants-3274151-supplementary.pdf]

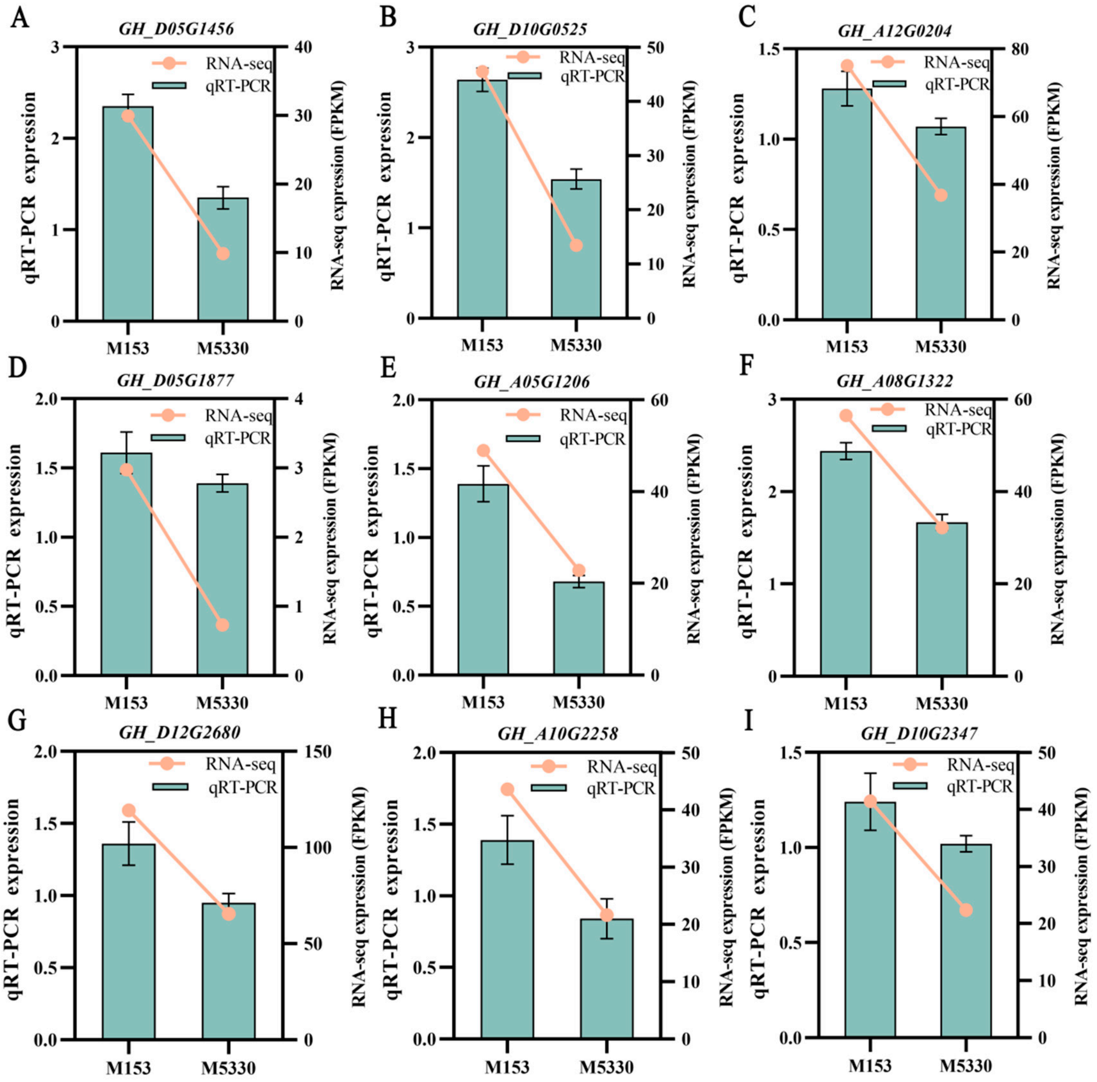

Figure S1. Analysis of RNA-seq data reliability.

Table S2. List of the EDGs relatede to the phenylpropanoid biosynthesis pathway.

Gene ID	|Fold Change|	Annotation
GH_A10G2258	2.0161	*Phenylalanine ammonia lyase* (*PAL*)
GH_D10G2347	1.8513	*Phenylalanine ammonia lyase* (*PAL*)
GH_A04G0918	3.0610	*Phenylalanine ammonia lyase* (*PAL*)
GH_A10G0499	2.6716	*4-coumarate:CoA ligase* (*4CL*)

Note: The DEGs relatede to the phenylpropanoid biosynthesis pathway were identified by using the criteria of fold change ≥1.5 and False Discovery Rate (FDR) < 0.05.

GH_A05G1439	4.8302	*4-coumarate:CoA ligase* (*4CL*)
GH_D05G1456	3.0377	*4-coumarate:CoA ligase* (*4CL*)
GH_D10G0525	3.3835	*4-coumarate:CoA ligase* (*4CL*)
GH_D09G1580	39.4751	*4-coumarate:CoA ligase* (*4CL*)
GH_D05G1877	4.0779	*Cinnamyl-alcohol dehydrogenase* (*CAD*)
GH_A12G0204	2.0401	*Cinnamyl-alcohol dehydrogenase* (*CAD*)
GH_A02G1628	1.8602	*Cinnamyl-alcohol dehydrogenase* (*CAD*)
GH_D12G0217	1.9147	*Cinnamyl-alcohol dehydrogenase* (*CAD*)
GH_A07G0038	2.3990	*Catechol-O-Methyltransferase* (*COMT*)
GH_A12G2656	1.6452	*Catechol-O-Methyltransferase* (*COMT*)
GH_A12G2614	3.6747	*Catechol-O-Methyltransferase* (*COMT*)
GH_D09G1239	2.3192	*Catechol-O-Methyltransferase* (*COMT*)
GH_D12G2680	1.8229	*Catechol-O-Methyltransferase* (*COMT*)
GH_A08G1322	1.7542	*Catechol-O-Methyltransferase* (*COMT*)
GH_D05G1208	1.6745	*Cinnamoyl-CoA reductase* (*CCR*)
GH_A05G1206	2.1409	*Cinnamoyl-CoA reductase* (*CCR*)
GH_A11G0457	2.5445	*Ferulate 5-hydroxylase* (*F5H*)
GH_D11G1903	8.6545	*Ferulate 5-hydroxylase* (*F5H*)
GH_A11G1870	68.4159	*Ferulate 5-hydroxylase* (*F5H*)
GH_D11G0478	3.8312	*Ferulate 5-hydroxylase* (*F5H*)
GH_D11G2500	5.9093	*Cinnamate 4-hydroxylase* (*C4H*)
GH_A11G2478	15.6436	*Cinnamate 4-hydroxylase* (*C4H*)

Gene ID	|Fold Change|	Annotation

Table S3. List of the EDGs relatede to the photosynthesis pathway.

GH_A11G1525	4.1473	*ATPB*
GH_A09G1192	2.0068	*ATPB*
GH_D05G3822	4.0656	*CAB13*
GH_A01G0665	5.9689	*CAB13*
GH_D01G0664	5.0214	*CAB13*
GH_A04G0248	4.8468	*CAB13*
GH_A01G1741	1.6955	*CAB7*
GH_A12G1680	1.5707	*CAB7*
GH_D01G1769	1.6931	*CAB7*
GH_D12G1682	2.0563	*CAB7*
GH_A10G0400	7.0633	*CAB21*
GH_A05G1509	12.6391	*CAB21*
GH_D10G0420	6.8757	*CAB21*
GH_D05G1535	23.3198	*CAB21*
GH_A07G1924	2.9043	*CAB151*
GH_D07G1817	2.5339	*CAB151*
GH_A07G2174	2.3752	*CAB151*
GH_A07G1831	3.3683	*CAB151*
GH_D02G2135	3.8064	*CAP10A*
GH_A03G1965	2.6875	*CAP10A*
GH_A13G0242	3.1946	*LHCA1*
GH_D13G0237	3.9294	*LHCA1*
GH_A05G0880	3.8987	*LHCA1*
GH_D05G0870	2.1160	*LHCA1*
GH_A11G1535	2.0330	*LHCA3*
GH_D11G1560	2.3155	*LHCA3*
GH_A07G0736	5.5803	*LHCA4*
GH_D07G0728	5.3806	*LHCA4*
GH_D12G1968	3.8944	*LHCA4*
GH_D01G2541	2.0523	*LHCB4*
GH_A01G2465	2.0180	*LHCB4*
GH_D05G2510	2.3711	*LHCB5*
GH_A05G2492	2.7575	*LHCB5*
GH_D03G0877	3.2382	*PSAA*
GH_D12G0041	2.4124	*PSAD*
GH_A12G0041	1.6157	*PSAD*
GH_A08G0728	4.6106	*PSAD*
GH_D08G0721	3.5071	*PSAD*
GH_A06G1621	1.8777	*PSAF*
GH_D06G1658	1.9097	*PSAF*
GH_A04G0007	1.8322	*PSAG*

GH_D02G2593	1.5134	*PSAK*
GH_A07G0991	3.4986	*PSAL*
GH_D07G0979	4.8226	*PSAL*
GH_A11G2422	1.5453	*PSAN*
GH_D07G1195	2.5518	*PSAO*
GH_A05G0811	2.5026	*PSAO*
GH_D05G0804	1.7516	*PSAO*
GH_A01G0171	1.6125	*PSB27*
GH_D01G1168	3.7534	*PSBB*
GH_A09G0617	3.0587	*PSBB*
GH_A12G2486	4.0410	*PSBB*
GH_D03G0873	2.4939	*PSBC*
GH_D08G1324	4.3138	*PSBD*
GH_A11G1964	1.5572	*PSBO*
GH_D10G1250	2.0786	*PSBP3*
GH_A10G1654	2.1702	*PSBP3*
GH_D10G0173	2.2200	*PSBQ*
GH_A06G1372	2.1773	*PSBQ*
GH_D06G1409	1.5726	*PSBQ*
GH_A10G0169	2.2052	*PSBQ*
GH_A11G2796	2.6883	*PSBW*

Note: The DEGs relatede to the photosynthesis pathway were identified by using the criteria of fold change ≥1.5 and False Discovery Rate (FDR) < 0.05.

Table S4. List of the EDGs relatede to the starch and sucrose biosynthesis pathways.

Gene ID	|Fold Change|	Annotation
GH_A13G2184	5.0856	*Sucrose Synthase* (*SUS*)

GH_A11G0433	2.7681	*Sucrose Synthase* (*SUS*)
GH_D06G0851	17.1847	*Sucrose Synthase* (*SUS*)
GH_D08G1434	1.5544	*Sucrose Synthase* (*SUS*)
GH_A05G0363	1.8286	*Sucrose Synthase* (*SUS*)
GH_D05G0367	2.0815	*Sucrose Synthase* (*SUS*)
GH_A10G2556	1.8971	*Sucrose Synthase* (*SUS*)
GH_A10G2551	1.7980	*Sucrose Synthase* (*SUS*)
GH_A10G2552	2.8015	*Sucrose Synthase* (*SUS*)
GH_D11G3434	2.4633	*Sucrose Synthase* (*SUS*)
GH_A05G2554	1.7972	*Amylase* (*AMY*)
GH_D01G1246	2.0497	*Amylase* (*AMY*)
GH_A05G2029	2.5747	*Amylase* (*AMY*)
GH_D01G1247	2.5203	*Amylase* (*AMY*)
GH_D05G2061	1.8411	*Amylase* (*AMY*)
GH_A01G1180	3.0281	*Amylase* (*AMY*)
GH_A09G2029	2.5747	*WAXY*
GH_D11G2097	2.6941	*Invertase* (*INV*)
GH_A06G0039	1.8161	*Hexokinase* (*HK*)
GH_A07G2210	1.5920	*Glucose-1-phosphate* (*G1P*)

Note: The DEGs relatede to the starch and sucrose biosynthesis pathways were identified by using the criteria of fold change ≥1.5 and False Discovery Rate (FDR) < 0.05.

Table S5. List of the EDGs relatede to the cellulose synthesis pathways.

Gene ID	|Fold Change|	Annotation
GH_A07G2317	11.8174	*Cellulose Synthase A* (*CesA4*)

GH_A08G0515	11.0563	*Cellulose Synthase A* (*CesA4*)
GH_D08G0525	12.9380	*Cellulose Synthase A* (*CesA4*)
GH_D07G2262	17.7536	*Cellulose Synthase A* (*CesA4*)
GH_D07G0439	23.0240	*Cellulose Synthase A* (*CesA4*)
GH_A07G0437	14.3332	*Cellulose Synthase A* (*CesA7*)
GH_D05G0094	37.6259	*Cellulose Synthase A* (*CesA7*)
GH_A05G0089	19.5501	*Cellulose Synthase A* (*CesA7*)
GH_D05G1574	4.0522	*Cellulose Synthase A* (*CesA8*)
GH_A10G0369	37.4603	*Cellulose Synthase A* (*CesA8*)
GH_D10G0384	37.2205	*Cellulose Synthase A* (*CesA8*)
GH_D13G1867	1.8878	*Cellulose Synthase E* (*CesE6*)
GH_A09G2447	1.7777	*Cellulose synthase-like* (*CslG3*)
GH_A12G2574	8.0419	*Cellulose synthase-like* (*CslG3*)

Note: The DEGs relatede to the cellulose synthesis pathways were identified by using the criteria of fold change ≥1.5 and False Discovery Rate (FDR) < 0.05.
